# Efficient expression of sortase A from *Staphylococcus aureus* in *Escherichia coli* and its enzymatic characterizations

**DOI:** 10.1186/s40643-017-0143-y

**Published:** 2017-02-18

**Authors:** Zhimeng Wu, Haofei Hong, Xinrui Zhao, Xun Wang

**Affiliations:** 1 The Key Laboratory of Carbohydrate Chemistry and Biotechnology, Ministry of Education, School of Biotechnology, Jiangnan University, 1800 Lihu Road, Wuxi, China; 2 State Key Laboratory of Natural and Biomimetic Drugs, Peking University, Beijing, 100191 China

**Keywords:** Sortase A, *Escherichia coli*, Response surface methodology, 7-L fermentor, Enzymatic characterizations

## Abstract

**Background:**

Sortase A (SrtA) is a transpeptidase found in *Staphylococcus aureus*, which is widely used in site-specific protein modification. However, SrtA was expressed in *Escherichia coli* (*E. coli*) in rather low level (ranging from several milligrams to 76.9 mg/L at most). The present study aims to optimize fermentation conditions for improving SrtA expression in *E. coli*.

**Results:**

Under the optimized media (0.48 g/L glycerol, 1.37 g/L tryptone, 0.51 g/L yeast extract, MOPS 0.5 g/L, PBS buffer 180 mL/L) and condition (30 °C for 8 h) in a 7-L fermentor, the enzyme activity and the yield of SrtA reached 2458.4 ± 115.9 U/mg DCW and 232.4 ± 21.1 mg/L, respectively, which were higher by 5.8- and 4.5-folds compared with initial conditions, respectively. The yield of SrtA also represented threefold increase than the previously reported maximal level. In addition, the enzymatic characterizations of SrtA (optimal temperature, optimal pH, the influence of metal irons, and tolerance to water-soluble organic solvents) were determined.

**Conclusions:**

Enhanced expression of SrtA was achieved by optimization of medium and condition. This result will have potential application for production levels of SrtA on an industry scale. Moreover, the detailed enzymatic characterizations of SrtA were examined, which will provide a useful guide for its future application.

**Electronic supplementary material:**

The online version of this article (doi:10.1186/s40643-017-0143-y) contains supplementary material, which is available to authorized users.

## Background

Sortase A (SrtA, EC number: 3.4.22.70) is a membrane-anchored transpeptidase first found in *Staphylococcus aureus*, which anchors surface proteins to cell wall by a cell-wall sorting reaction (Mazmanian et al. 1999). It recognizes a specific sorting signal peptide (Leu-Pro-X-Thr-Gly, with X standing for any amino acid except cysteine) at the C-terminal of target proteins, and cleaves the amide bond between threonine and glycine to generate a thioester intermediate; then the intermediate reacts with the amino group of pentaglycine cross-bridges resulting in the attachment of proteins to cell surface (Perry et al. 2002a, b). SrtA is a promiscuous enzyme, which can accept a variety of oligoglycine-modified nucleophiles as its substrate (Bentley et al. 2008). In addition, the truncated SrtA (Δ59 SrtA), by removing its original N-terminal transmembrane domain, showed good water solubility and retained the same transpeptidation activity as full-length SrtA (Ilangovan et al. 2001). Therefore, in recent years, SrtA-mediated ligation (SML) was widely used in protein-to-protein fusions (Witte et al. 2012), peptide and protein cyclizations (Wu et al. 2011), immobilization of biocatalyst onto solid surface (Chan et al. 2007), preparation of complex glycoconjugates (Guo et al. 2009), antibody–drug conjugation (Beerli et al. 2015,Voloshchuk et al. 2015), and in vivo protein modification (Glasgow et al. 2016).

With the development of SML, the demand for high-level expression of SrtA is very high. *E. coli* are the most studied expression system for SrtA production because of its effective genetic manipulation and cheap culturing cost (Vincentelli and Romier 2013). Since the recombinant Δ59 SrtA was first achieved in 1999 using pQE30 (Qiagen) as vector in *E. coli* (Ton-That et al. 1999), several groups have cloned and expressed SrtA using several commercial plasmids, including pET23b (Novagen) (Guo et al. 2009), pTWIN1 (New England Biolabs) (Bentley et al. 2008) and pBAD (Invitrogen) (Kim et al. 2002), etc., with slightly modified protocols. However, the reported yield of SrtA varied differently and remained at low level ranging from several milligrams to the maximum of 76.9 mg/L (Kruger et al. 2004). In our ongoing studies on SML for the synthesis of complex glycoconjugates (Wu et al. 2010, 2013), we realized that the low-level production of SrtA is difficult to meet the requirement of future industrial applications. Therefore, more endeavors should be made to improve the expression level of this important enzyme.

Heterologous expression of recombinant protein in *E. coli* is influenced by many factors, including medium composition, induction temperature, initial pH, and so on (Lee et al. 1997). In this study, Δ59 SrtA was cloned and successfully expressed in *E. coli* at first. Then, the optimization of fermentation conditions for SrtA production was manipulated by the combination of traditional one-factor-at-a-time approach and response surface methodology (RSM) at shake-flask stage, which is an effective and simplified method that has gained great success in the production of recombinant proteins (Papaneophytou and Kontopidis 2014). Finally, high-level expression of Δ59 SrtA was achieved in a 7-L fermentor, and the enzymatic characterizations of SrtA were examined.

## Methods

### Strain, plasmid, and media

The host strain *E. coli* BL21 (DE3) and plasmid pET28a were purchased from Novagen (Madison, WI). Luria broth (LB) medium (Tryptone 10 g/L, Yeast extract 5 g/L, NaCl 10 g/L), Terrific broth (TB) medium (Tryptone 12 g/L, Yeast extract 24 g/L, Glycerol 4 g/L) with 100 mL/L PBS buffer (K_2_HPO_4_·3H_2_0 164.3 g/L, KH_2_PO_4_ 23.1 g/L), Super broth (SB) medium (Tryptone 30 g/L, Yeast extract 20 g/L, 3-(N-Morpholino) propanesulfonic acid (MOPS) 10 g/L), Soybean–peptone–yeast extract broth (SOB) medium (Tryptone 20 g/L, Yeast extract 5 g/L, NaCl 0.5 g/L, KCl 0.2 g/L) and 2× Yeast extract/tryptone (2× YT) medium (Tryptone 16 g/L, Yeast extract 10 g/L, NaCl 5 g/L), were used to perform the expression of SrtA, respectively.

### Expression of SrtA in *E. coli* BL21 (DE3)

The genome of *S. aureus* was extracted by Genomic Extraction Kit (Qiagen, Valencia, CA, USA) and applied as the template for amplifying Δ59-*srtA* with the primer pairs: CAT GCC ATG GAA GCT AAA CCT CAA ATT CCG; and CGC GGA TCC TTA GTG GTG GTG ATG ATG ATG TTT GAC TTC TGT AGC TAC AAA GAT. The gel-purified PCR-amplified Δ59-*srtA* fragments were digested and inserted into the *Nco*I/*Bam*HI site of pET28a. The plasmid (pET28a-Δ59-srtA) confirmed by DNA sequencing was transformed into *E. coli* BL21 (DE3) strain. The positive clones were propagated in LB medium at 37 °C overnight with constant shaking at 200 rpm. The seed culture (2%) was inoculated into 25 mL fermentation medium at 37 °C until the OD_600_ reached 0.6 and then incubated with 1 mM IPTG (isopropyl β-d-thiogalactoside) at 25 °C for 8 h. When the fermentation was completed, cells were pelletized and ultrasonicated by a probe VCX800 system (Sonics, Newtown, USA). The lysate supernatant were collected for the activity assay and purification.

### SrtA activity assay

The specific substrate of SrtA (Dabcyl-QALPETGEE-Edans) was obtained from GL biochem Ltd. (Shanghai, China). The SrtA activity arrays were performed in 200-μL volume of 50 mM Tris–HCl buffer (including 150 mM NaCl, 30 mM CaCl_2_, 0.5 mg Dabcyl-QALPETGEE-Edans, pH 7.8), and 10 μL SrtA lysate supernatant. The reactions were carried out at 37 °C for 1 h by means of a Synergy H4 hybrid microplate reader (BioTek, Vermont, America), and the fluorescence intensity (FI) was detected with 350 nm for excitation and 495 nm for recordings. One unit of SrtA activity was defined as the amount of enzyme (mg) that was able to increase at the rate of one FI per minute in the 200-μL reaction mixture.

### Purification of SrtA

The lysate supernatant and Ni–NTA agarose (Qiagen, Hilden, Germany) were loaded onto a gravity-flow column and incubated for 4 h at 4 °C. Then, the agarose was washed with a stepwise gradient of imidazole (10–40 mM) to eliminate intracellular contaminating proteins. The C-terminal His-tagged SrtA was eluted from the column using 500 mM imidazole and desalted using an Amicon Ultra 3 K device (Millipore, Billerica, USA). The concentration of purified SrtA was determined by the Bradford method and was used to calculate the yield of SrtA for each sample.

### Single-factor optimization

The optimal medium and several important fermentation conditions (induction time, induction temperature, and initial pH) for SrtA production were obtained by single factor optimization in 250-mL shaking flasks containing 25 mL of sterilized medium. The concentrations of alternative carbohydrates and nitrogenous compounds were equivalent to the concentrations of carbon and nitrogen resources in the initial medium (12 g/L tryptone; 4 g/L glycerol). In each experiment, one factor was changed, while the other factors were held constant. After fermentation, cell growth was monitored by measuring OD_600_ and correlated it with dry cell weight (DCW); the intracellular SrtA activity was measured by the method described in SrtA activity assay. Each experiment was performed in triplicate for the biological replicates, and the average values of enzymatic activity and biomass were used to select the optimal medium and conditions.

### Plackett–Burman Design

In order to enhance SrtA production, it is necessary to select those variables with major effects at first. Applying Plackett–Burman Design, fractional two-level factorial designs can be used for the efficient and economical screening. Experimental design was formulated according to the Plackett–Burman Design tool of Design Expert 7.0 software (Statease, USA) for the selection of significant factors. Nine factors were selected (the concentration of glycerol, tryptone, yeast extract, PBS buffer, and MOPS in the medium, induction occasion, the concentration of IPTG, inoculation, loading volume), each of which was coded with two levels (Table 1). The twelve experiments were performed in shaking-flask fermentation under conditions fixed by single factor optimization (all cultivations were carried out in 250 mL shake flasks at 200 rpm). All experiments were performed in triplicates, and the average values of enzymatic activity and biomass were used to select the optimal medium and conditions. The *P* values were calculated by Duncan’s multiple range tests. The optimal values of non-significant factors were determined by the prediction tool of Design Expert 7.0.

**Table 1:** The Plackett–Burman design and effects of nine factors for SrtA production

Factors	Code	High level (+1)	Low level (−1)	Coefficient	*F* value	*P* value
Glycerol (g/L)	A	1.0	0.1	−162.08	27.97	0.03
Tryptone (g/L)	B	4.0	1.0	−243.91	63.35	0.02
Yeast extract (g/L)	C	4.0	1.0	−201.77	43.35	0.02
PBS (%)	D	20.0	5.0	−17.02	0.31	0.63
MOPS (g/L)	E	1.5	0.5	−85.82	7.84	0.11
Induction occasion (OD_600_)	F	1.0	0.4	113.59	13.74	0.07
IPTG (mM)	G	1.5	0.2	33.43	1.19	0.39
Inoculation (%)	H	5.0	1.0	−4.80	12.83	0.89
Loading volume (%)	I	20.0	6.0	−109.76	0.03	0.07

### Box–Behnken Design

Box and Behnken Design can propose three level designs for fitting response surfaces to get the best values for different variables by second-order polynomial model. Therefore, the concentration of three significant factors (glycerol, tryptone, and yeast extract) in the medium was optimized by the Box–Behnken Design tool of Design Expert 7.0 software. Each factor was coded with three levels (Additional file 1: Table S1) and seventeen experiments were performed in shaking-flask fermentation under previous fixed conditions (all cultivations were carried out in 250 mL shake flasks at 200 rpm). All experiments were performed in triplicates, and the average values of enzymatic activity and biomass were used to select the optimal medium and conditions. The *P* values were calculated by Duncan’s multiple range tests. The optimal concentrations of glycerol, tryptone, and yeast extract were determined by the prediction tool of software.

### The enzymatic characterizations of SrtA

The effect of pH on SrtA activity was measured in the 200 μL reaction system at 37 °C (150 mM NaCl, 10 mM CaCl_2_, 0.5 mg Dabcyl-QALPETGEE-Edans) in a pH range of 3.0–11.0, using the appropriate buffers at concentration of 50 mM (3.0–5.0, sodium citrate; 6.0–8.0, sodium phosphate; 8.0–9.0, Tris–HCl; 10.0-11.0, and NaHCO_3_–NaOH). The optimal temperature for SrtA activity was determined at various temperatures ranging from 20 to 80 °C. To determine the effect of metal ions on SrtA activity, the enzyme assays were measured without additional metal ion (control) or with 5 mM different metal ions (Ca^2+^, Mn^2+^, Mg^2+^, Co^2+^, Cu^2+^, Fe^2+^, Ni^2+^ and Zn^2+^). As for the optimal concentration of Ca^2+^, the SrtA activities were detected at a concentration ranging from 1 mM to 100 mM. At last, the effect of soluble organic solvents (acetone, methanol, ethanol, acetonitrile, and dimethyl sulfoxide) on SrtA activity were determined, the enzyme assays were measured at a content of ranging from 10 to 50%. All experiments were performed in triplicate, and the mean values were used for calculations.

## Results and discussion

### Selection of the components of fermentation medium by single factor optimization

**Fig. 1:**
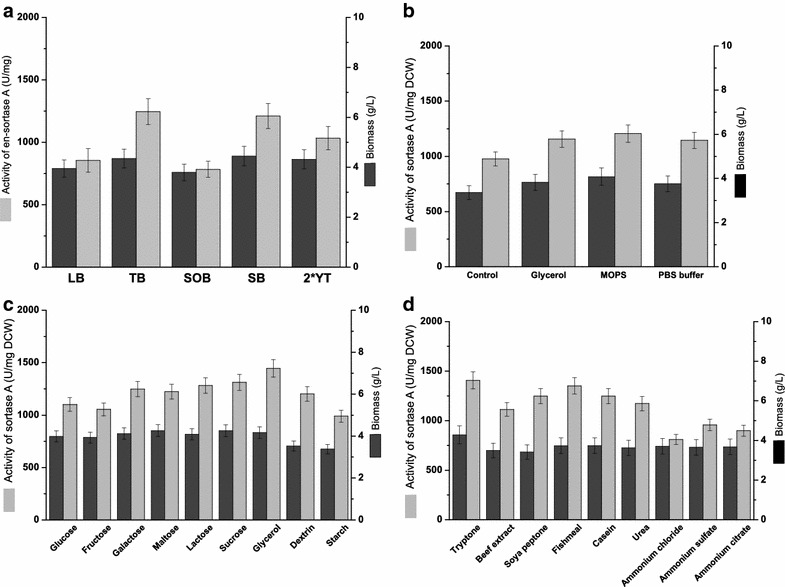
**a** Effects of media on the cell growth and SrtA expression. *Light gray* and *dark gray bars* represent the SrtA activity and biomass in mediums of LB, TB, SOB, SB and 2×YT.** b** Effects of addition of defined compositions in media on the expression of SrtA. *Light gray* and *dark gray bars* represent the SrtA activity and biomass in the TB medium with addition of glycerol, MOPS, and PBS buffer. The TB medium without glycerol and PBS buffer was used as the control. **c** Effects of various carbon sources on the expression of SrtA. *Light gray* and *dark gray bars* represent the SrtA activity and biomass in the medium with various carbon sources of glucose, fructose, galactose, maltose, lactose, sucrose, glycerol, dextrin, and starch. **d** Effects of nitrogen sources on the expression of SrtA. *Light gray* and *dark gray bars* represent the SrtA activity and biomass in the medium with various nitrogen sources of tryptone, beef extract, soya peptone, fishmeal, casein, urea, ammonium chloride, ammonium sulfate, and ammonium citrate

Fermentation medium has a profound influence on the expression of recombinant proteins in *E. coli* among many factors [Tseng and Leng 2012, Li et al (2014)]. Thus, starting from the *E. coli* BL21 (DE3) strain encoding pET28a-Δ59-srtA plasmid, five media (LB, TB, SOB, SB and 2×YT) were screened to express SrtA at shaking-flask level at 37 °C for 4 h in an initial experiment. As shown in Fig. 1a, when expressed in TB and SB media, the SrtA activity values were 1245.2 and 1210.7 U/mg DCW, respectively, which were higher than those in LB, SOB, and 2×YT media (855.2, 783.2 and 1032.9 U/mg DCW, respectively). This result indicated that using the rich media improved the expression level of SrtA. By comparing the medium compositions of TB and SB, defined components may affect the SrtA expression level. Therefore, glycerol, PBS buffer, and MOPs were added into the control media (12 g/L tryptone and 24 g/L yeast extract), which were used as fermentation medium in SrtA expression, respectively. It was interesting to observe that the addition of glycerol and PBS enhanced the enzymatic activities by 18.3 and 17.3%, respectively. Addition of MOPS increased the SrtA activity by 23.5% (Fig. 1b). Thus, we choose TB medium (12 g/L tryptone, 24 g/L yeast extract, 4 g/L glycerol, and 100 mL/L PBS buffer) with the addition of 10 g/L MOPS as the initial SrtA fermentation medium.

Because both carbon and nitrogen sources are important nutrients in the media for protein expression (Scott et al. 2002), these two nutrients were screened to find the optimal alternatives for SrtA expression. Five different types of carbohydrates (glucose, fructose, galactose, maltose, lactose, sucrose, dextrin, and starch) and nine nitrogenous compounds (beef extract, soya peptone, fishmeal, casein, urea, ammonium chloride, ammonium sulfate, and ammonium citrate) were used to replace the primary carbon and nitrogen sources in TB medium. As shown in Fig. 1c, glycerol was the suitable carbon source for SrtA expression which showed the best strain growth and enzymatic activity (1446.7 U/mg DCW). As for the nitrogen source, organic nitrogen source was superior to inorganic nitrogen source. As Fig. 1d shows, tryptone was the most suitable nitrogen source for SrtA expression, which gave the highest values of cell biomass and enzymatic activity (1407.1 U/mg DCW). Hence, glycerol and tryptone were selected in the subsequent experiments.

### Influences of induction time, induction temperature, and initial pH on SrtA production

**Fig. 2:**
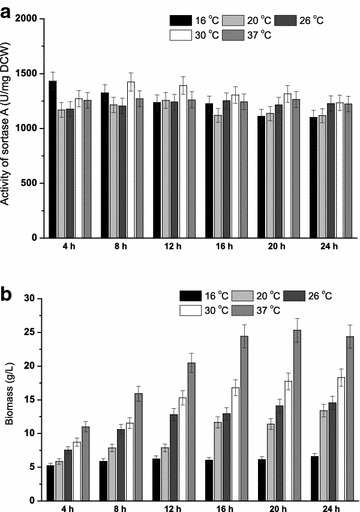
Effects of induction time and induction temperature on the expression of SrtA. **a** The SrtA activities under various induction times and induction temperatures; **b** the biomasses under various induction times and induction temperatures

**Fig. 3:**
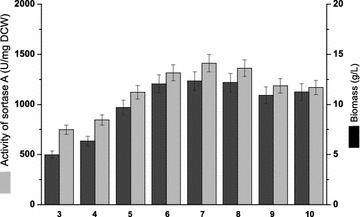
Effect of initial pH on the expression of SrtA. *Light gray* and *dark gray bars* represent the SrtA activities and biomasses in the medium with different initial pH values

After the fermentation medium was optimized, the induction time, induction temperature, and initial pH were revisited as these parameters varied greatly in previous reports (Ton-That et al. 1999; Hirakawa et al. 2012; Lee et al. 2002). In previous study on SrtA expression, higher induction temperature (37 °C) (Ilangovan et al. 2001) and shorter induction time (within 6 h) (Kim et al. 2002) were mostly applied. However, it was found that the lower induction temperature (below 30 °C) (Tanaka et al. 2008) and longer induction time (more than 8 h) (Matsushita et al. 2009) is helpful for SrtA production. In this study, induction time (4, 8, 12, 16, 20 and 24 h) and induction temperature (16, 20, 26, 30 and 37 °C) were combined to perform shaking-flask fermentation (Fig. 2). The highest SrtA activity (1431.3 U/mg DCW) was obtained at 16 °C for 4 h (Fig. 2a), but the strain growth was extremely poor under this condition (Fig. 2b). Therefore, to maintain sufficient cell density for SrtA expression, fermentation at 30 °C for 8 h (the second highest SrtA activity) was chosen as the optimal condition in the following experiments. As for initial pH, another important factor for the heterogeneous expression in *E. coli* (Buchanan and Klawitter 1992), strain growth was inhibited when pH was below 6.0 or above 9.0; while SrtA activity reached the highest enzymatic activity (1411.4 U/mg DCW) when pH was 7.0 (Fig. 3). Therefore, the optimal initial pH was chosen at pH 7.0.

### Determination of significant factors for SrtA production by Plackett–Burman design

Besides the optimal parameters mentioned above, there are still many factors that influence SrtA production. Based on the SrtA activity of each experiment designed by the principle of Plackett–Burman, the effect of these factors were evaluated (Additional file 1: Table S2). The fitted equation (in coded value) was obtained from the twelve tests based on the first-order model:

$$Y \, ({\text{sortase A activity}}) = 645.67 - 162.08 \times {\text{A }} - 243.91 \times {\text{B}} - 201.77 \times {\text{C}} - 17.02 \times {\text{D}} - 85.82\times {\text{E}} + 113.59 \times {\text{F}} + 33.43 \times {\text{G}}- 4.80 \times {\text{H}}-109.76 \times {\text{I}}$$

The coefficient of determination (*R* ^2^ = 0.9884) indicates that 98.84% of model terms satisfy the model, and the fitting degree of the regression equation is good. The results revealed that the concentration of glycerol, tryptone, yeast extract are the significant factors (*P* value <0.05) and the other six factors have weaker effect (*P* value >0.05). Forecasted by Design-Expert software, the value of these six nonsignificant factors are defined as IPTG 1.5 mM, MOPS 0.5 g/L, PBS buffer 180 mL/L, induction occasion (OD_600_) 1.0, inoculum 2%, and loading volume 10%. Furthermore, the negative coefficients of glycerol (−162.08), tryptone (−243.91), and yeast extract (−201.77) indicated that the low concentrations of glycerol, tryptone, and yeast extract were beneficial for SrtA production.

### Box–Behnken design results for the significant factors

In the following, Box–Behnken design was applied to refine the optimal levels of three selected significant factors (glycerol, tryptone, and yeast extract) for SrtA production. The high levels of glycerol, tryptone, and yeast extract were maintained at 4.0 g/L, while their low levels were reduced into 0.5 g/L. Seventeen experiments were performed in shaking-flask fermentation under previously fixed conditions (Table 2). The experimental data were fitted to obtain the second-order polynomial model (in coded value) equation:

**Table 2:** The Box–Behnken design matrix and experimental results

Runs	A	B	C	SrtA production (U/mg DCW)
1	0	0	0	1491.1
2	0	−1	+1	1394.1
3	+1	−1	0	971.6
4	+1	0	+1	912.2
5	−1	−1	0	1418.7
6	0	0	0	1468.3
7	0	+1	+1	1505.3
8	−1	0	−1	1666.6
9	0	+1	−1	1588.2
10	+1	0	−1	988.1
11	−1	+1	0	1427.9
12	+1	+1	0	974.5
13	0	0	0	1464.8
14	0	−1	−1	1566.6
15	−1	0	+1	1457.3
16	0	0	0	1492.4
17	0	0	0	1482.4

$$Y \, ({\text{sortase A activity}}) = 1479.80 + 18.11 \times {\text{A}}- 265.51 \times {\text{B}}- 67.58 \times {\text{C}}- 1.5 7 \times {\text{AB}}+ 22.40 \times {\text{AC}}+ 33.35 \times {\text{BC}} -12.06 \times {\text{A}}^{2} -269.56 \times {\text{B}}^{ 2} +45.81 \times {\text{C}}^{ 2}$$

**Fig. 4:**
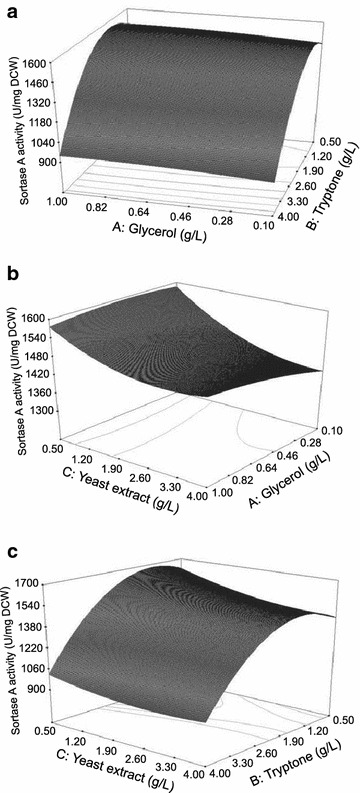
The response 3D surface plots of three significant factors. **a** Glycerol with tryptone; **b** glycerol with yeast extract; **c** Tryptone with yeast extract. The response 3D surface plots were obtained using the Design Expert 7.0 software directly

The results of ANOVA (analysis of variance) are summarized in Additional file 1: Table S3. The value of *R* ^2^ is 0.9833, suggesting that 98.33% of model terms satisfy the model and the equation was suitable for representing the experimental data. Compared with the Plackett–Burman design results, the Box–Behnken design results further confirmed the significant negative effects of tryptone and yeast extracts on the SrtA production (both *P* values <0.01), while the effect of glycerol was not apparent (*P* value >0.05). The interaction of the three factors were further analyzed, and the 3D surface plots are shown in Fig. 4. Based on the credible model equation, the maximum SrtA production (1674 U/mg DCW predicted) could be obtained at 0.48 g/L glycerol, 1.37 g/L tryptone, and 0.51 g/L yeast extract.

**Fig. 5:**
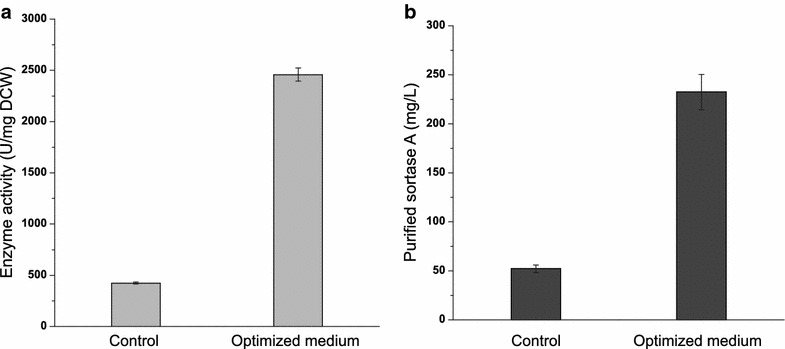
Validation of the optimal conditions for SrtA expression. The LB medium was used as the control at 37 °C for 4-h fermentation, while the optimized media (0.48 g/L glycerol, 1.37 g/L tryptone, 0.51 g/L yeast extract, MOPS 0.5 g/L, PBS buffer 180 mL/L) were operated at 30 °C for 8 h. **a** The SrtA activity in the control and optimized media at 7-L fermentor level; **b** the concentration of purified SrtA in the control and optimized media at 7-L fermentor level

To verify this model, SrtA expression was performed in a 7-L fermentor under the predicted optimal medium and conditions. The enzyme activity and yield of SrtA were 2458.4 ± 115.9 U/mg DCW and 232.4 ± 21.1 mg/L, respectively. Compared with the control (LB medium) and initial conditions (37 °C for 4 h), the enzyme activity and yield of SrtA were increased by 5.8- and 4.5-folds, respectively (Fig. 5a, b; Table 3). And to the best of our knowledge, this result represented the highest yield of SrtA expression ever reported, which is threefold increase compared with the highest production of SrtA in literature (76.9 mg/L) (Naik et al. 2006).

**Table 3:** The enzyme activity and yield of SrtA performed in the optimized conditions (optimized medium, 30 °C for 8 h) and initial conditions (LB medium, 37 °C for 4 h) with shaking-flask fermentation and at 7-L fermentor level

Samples	Shaking-flask level		7-L fermentor level	
	Activity (U/mg DCW)	Yield (mg/L)	Activity (U/mg DCW)	Yield (mg/L)
Initial conditions	855.2 ± 95.1	44.2 ± 2.1	423.9 ± 8.8	52.1 ± 3.9
Predicted values	1674	155	–	–
Optimized conditions	1732.3 ± 122.4	188.1 ± 18.3	2458.4 ± 115.9	232.4 ± 21.1

### The enzymatic characterizations of Srt A

**Fig. 6:**
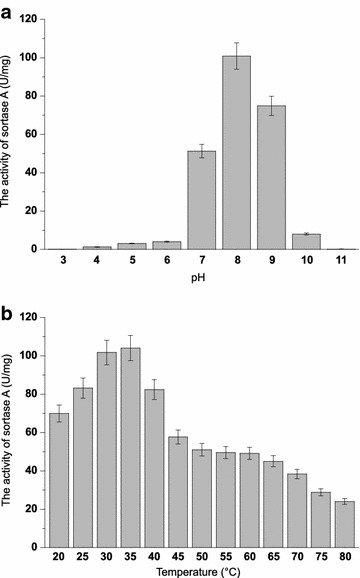
The optimal pH and temperature for SrtA. **a** Effect of pH on SrtA activity under a pH range of 3–11; **b** effect of temperature on SrtA activity at various temperatures ranging from 20 to 80 °C

**Fig. 7:**
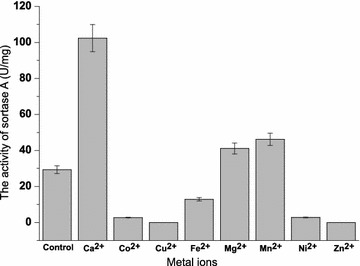
Effects of metal ions (5 mM) on SrtA activity. Effect of metal ions on SrtA activity. The enzyme assays were performed in the absence (control) and the presence of 5 mM various metal ions of Ca^2+^, Co^2+^, Cu^2+^, Fe^2+^, Mg^2+^, Mn^2+^, Ni^2+^, and Zn^2+^

**Fig. 8:**
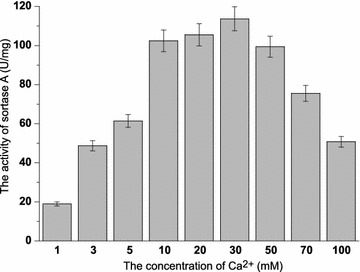
Effect of Ca^2+^ concentration on SrtA activity. Effect of Ca^2+^ concentration on SrtA activity in the range of 1–100 mM Ca^2+^

**Fig. 9:**
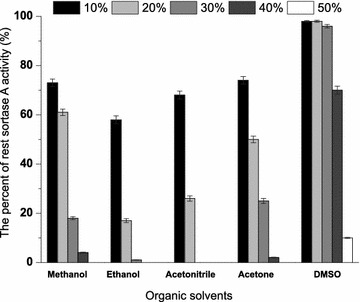
Effect of organic solvents on SrtA activity. Effects of organic solvents on SrtA activity in the presence of 10–50% of methanol, ethanol, acetonitrile, acetone, and DMSO

SrtA-mediated ligation has been widely used in site-specific protein modification in recent years. However, to our knowledge, the enzymatic characterizations of SrtA were explored incompletely. These parameters are particularly important for the application of SrtA in ligation reactions. The optimal pH of SrtA activity was measured from pH 3–11 using FRET substrate Dabcyl-QALPETGEE-Edans. SrtA retained good activity over a pH range of 7.0–9.0 and displayed the optimal activity at pH 8.0 (Fig. 6a). For thermostability, SrtA remained its 50% of activity over a range of 20–60 °C and the optimal temperature was 35 °C (Fig. 6b). SrtA was Ca^2+^-dependent enzyme. However, the influence of other metal irons (Co^2+^, Cu^2+^, Fe^2+^, Mg^2+^, Mn^2+^, Ni^2+^, and Zn^2+^) and the most suitable concentration of Ca^2+^ to the activity of SrtA were unknown. With regard to the influence of metal irons, although Ca^2+^ has been proven to be necessary for SrtA activity and 5–10 mM Ca^2+^ was added in previous SrtA-catalyzed reactions (Wu et al. 2013), the effects of other metal irons (such as Mn^2+^, Cu^2+^, Fe^2+^, etc.) on the catalytic efficiency of SrtA and the most suitable concentration of Ca^2+^ for SrtA activity were unknown. As shown in Fig. 7, Ca^2+^ could enhance the activity of SrtA dramatically, which is consistent with the literature data. The inhibition effect on SrtA in the presence of other metal ions in the decreasing order is Cu^2+^>Zn^2+^>Ni^2+^>Co^2+^>Fe^2+^>Mg^2+^>Mn^2+^. As for the optimal concentration of Ca^2+^, there was positive correlation between SrtA activity and calcium concentration to certain extent, and the best concentration of Ca^2+^ for SrtA activity is 30 mM (Fig. 8). Finally, organic solvents were frequently used as co-solvents to help in dissolving substrates in the SrtA-mediated reaction (Madej et al. 2012). The tolerance of SrtA to water-soluble organic solvents was examined. As shown in Fig. 9, SrtA is tolerant to 10% of methanol, ethanol, acetonitrile, and acetone; the enzyme activities remained at 72, 59, 70 and 73%, respectively; and SrtA lost its majority of activities with the increasing concentrations of these solvents to 30%. However, SrtA is well tolerant to dimethyl sulfoxide (DMSO) up to as high as 30% without any obvious activity loss. 40% of DMSO could decrease the enzyme activity to 70, and 50% of DMSO is detrimental to its activity.

## Conclusion

In summary, high-level expression of enzyme SrtA was achieved by a combination of single-factor optimization and response surface methodology in this study. Applying optimized medium (0.48 g/L glycerol, 1.37 g/L tryptone, 0.51 g/L yeast extract, MOPS 0.5 g/L, and PBS buffer 180 mL/L) and conditions (30 °C for 8 h), the highest enzyme activity and yield of SrtA could reach up to 2458.4 ± 115.9 U/mg DCW and 232.4 ± 21.1 mg/L, respectively. This formulation will have potential application of production levels of SrtA on an industry scale. In addition, the detailed enzymatic characterizations of SrtA were examined, which will provide a useful guide for its future application.

## Additional file

**Additional file 1.** Additional tables.
