# Supplementary material for: Efficient expression of sortase A from Staphylococcus aureus in Escherichia coli and its enzymatic characterizations
Source: Bioresour Bioprocess. 2017 Feb 18;4(1):13. doi: 10.1186/s40643-017-0143-y (PMC5316389; doi:10.1186/s40643-017-0143-y)
Supplement: Supplementary file 1 — Additional file 1. Additional tables. [file 40643_2017_143_MOESM1_ESM.docx]

**Supplementary Results** for **Efficient expression of sortase A from *Staphylococcus aureus* in *Escherichia* *coli* and its enzymatic characterizations**

Zhimeng Wu^a,b*^, Haofei Hong^a^, Xinrui Zhao^a^, Xun Wang^a^

Key Laboratory of Carbohydrate Chemistry & Biotechnology Ministry of Education,

School of Biotechnology, Jiangnan University, 1800 Lihu Avenue, Wuxi, Jiangsu, P.R.of China

[zwu@jiangnan.edu.cn](mailto:zwu@jiangnan.edu.cn)

**Table S1 The levels of glycerol, tryptone and yeast extract for Box-Behnken design**

| Factors | Code | High level (+1) | Center level (0) | Low level (-1) |
| --- | --- | --- | --- | --- |
| Glycerol (g/L) | A | 1.0 | 0.55 | 0.1 |
| Tryptone (g/L) | B | 4.0 | 2.5 | 0.5 |
| Yeast extract (g/L) | C | 4.0 | 2.5 | 0.5 |

**Table S2 The Plackett-Burman design matrix and experimental results**

| Runs | A | B | C | D | E | F | G | H | I | SrtA production  (U/mg DCW) |
| --- | --- | --- | --- | --- | --- | --- | --- | --- | --- | --- |
| 1 | +1 | - 1 | +1 | +1 | +1 | +1 | - 1 | - 1 | - 1 | 678.8 |
| 2 | +1 | +1 | - 1 | - 1 | +1 | - 1 | - 1 | +1 | - 1 | 334.1 |
| 3 | - 1 | +1 | - 1 | +1 | +1 | +1 | - 1 | +1 | +1 | 624.8 |
| 4 | - 1 | +1 | +1 | +1 | +1 | - 1 | +1 | - 1 | - 1 | 232.4 |
| 5 | +1 | - 1 | - 1 | - 1 | +1 | +1 | +1 | - 1 | +1 | 841.4 |
| 6 | - 1 | +1 | +1 | - 1 | - 1 | +1 | - 1 | - 1 | +1 | 443.6 |
| 7 | - 1 | - 1 | - 1 | +1 | - 1 | +1 | +1 | +1 | - 1 | 1577.8 |
| 8 | +1 | +1 | +1 | - 1 | - 1 | +1 | +1 | +1 | - 1 | 389.2 |
| 9 | +1 | +1 | - 1 | +1 | - 1 | - 1 | +1 | - 1 | +1 | 386.3 |
| 10 | - 1 | - 1 | - 1 | - 1 | - 1 | - 1 | - 1 | - 1 | - 1 | 1320.3 |
| 11 | - 1 | - 1 | +1 | - 1 | +1 | - 1 | +1 | +1 | +1 | 647.6 |
| 12 | +1 | - 1 | +1 | +1 | - 1 | - 1 | - 1 | +1 | +1 | 271.8 |

**Table S3 Analysis of variance (ANOVA) for SrtA production according to Box-Behnken design model**

| Source | Sum of squares | Degree of freedom | Mean square | Coefficient | F-value | P-value |
| --- | --- | --- | --- | --- | --- | --- |
| Model | 9.22*10^5^ | 9 | 1.03*10^5^ |  | 45.89 | < 0.01 |
| A | 2624.5 | 1 | 2624.5 | 18.11 | 1.18 | 0.31 |
| B | 5.64*10^5^ | 1 | 5.64*10^5^ | -265.51 | 252.55 | < 0.01 |
| C | 3.65*10^4^ | 1 | 3.65*10^4^ | -67.58 | 16.36 | < 0.01 |
| AB | 9.92 | 1 | 9.92 | -1.57 | 4.44*10^-3^ | 0.95 |
| AC | 2007.04 | 1 | 2007.04 | 22.40 | 0.90 | 0.37 |
| BC | 4448.89 | 1 | 4448.89 | 33.35 | 1.99 | 0.20 |
| A^2^ | 612.65 | 1 | 612.65 | -12.06 | 0.27 | 0.62 |
| B^2^ | 3.06*10^5^ | 1 | 3.06*10^5^ | -269.56 | 137.01 | < 0.01 |
| C^2^ | 8836.99 | 1 | 8836.99 | 45.81 | 3.96 | 0.09 |
| Residual | 1.56*10^4^ | 7 | 2233.10 |  |  |  |
| Lack of fit | 1.50*10^4^ | 3 | 4993.76 |  | 300.71 |  |
| Pure error | 650.46 | 4 | 162.62 |  |  |  |
| Correlational total | 9.38*10^5^ | 16 |  |  |  |  |
